# A new archaic baleen whale *Toipahautea waitaki* (early Late Oligocene, New Zealand) and the origins of crown Mysticeti

**DOI:** 10.1098/rsos.172453

**Published:** 2018-04-18

**Authors:** Cheng-Hsiu Tsai, R. Ewan Fordyce

**Affiliations:** 1 Department of Geology, University of Otago, PO Box 56, Dunedin 9054, New Zealand; 2 Department of Geology and Paleontology, National Museum of Nature and Science, 4-1-1 Amakubo, Tsukuba 305-0005, Japan; 3 Department of Paleobiology, National Museum of Natural History, Smithsonian Institution, PO Box 37012, Washington, DC 20013-7013, USA; 4 Department of Vertebrate Zoology, National Museum of Natural History, Smithsonian Institution, PO Box 37012, Washington, DC 20013-7013, USA

**Keywords:** Cetacea, mysticete, feeding strategy, filter-feeding, specialist/generalist, opportunist

## Abstract

A new genus and species of extinct baleen whale ^†^*Toipahautea waitaki* (Late Oligocene, New Zealand) is based on a skull and associated bones, from the lower Kokoamu Greensand, about 27.5 Ma (local upper Whaingaroan Stage, early Chattian). The upper jaw includes a thin, elongate and apparently toothless maxilla, with evidence of arterial supply for baleen. Open sutures with the premaxilla suggest a flexible (kinetic) upper jaw. The blowhole is well forward. The mandible is bowed laterally and slightly dorsally; unlike the Eomysticetidae, there are no mandibular alveoli, and the coronoid process is tapered and curved laterally. Jaw structure is consistent with baleen-assisted gulp-feeding. The age of early Chattian makes ^†^*Toipahautea* a very early, if not the oldest named, toothless and baleen-bearing mysticete, suggesting that the full transition from toothed to baleen-bearing probably occurred in the Early Oligocene. Late Oligocene mysticetes vary considerably in jaw form and kinesis, tooth form and function, and development of baleen, implying a wide range of raptorial, suctorial and filter-feeding behaviour. More study may elucidate the function of jaws, teeth and baleen in terms of opportunist/generalist feeding, as in modern gray whales, versus specialized feeding. We here propose that early mysticetes, when transitioned from toothed to baleen-bearing, were generalists and opportunists instead of specializing in any forms of feeding strategies. In addition, two different phylogenetic analyses placed ^†^*Toipahautea* either in a polytomy including crown Mysticeti, or immediately basal to the crown, and above †Eomysticetidae in both cases. Because the ^†^*Toipahautea waitaki* holotype is an immature individual, it may plot more basally in phylogeny than its true position.

## 1. Introduction

The origin of baleen and microphagous feeding by cetaceans marks a major evolutionary breakthrough, leading ultimately to the emergence of the largest animal, the blue whale *Balaenoptera musculus* (Cetacea: Mysticeti). Recent descriptions and interpretations of new fossil whales have greatly improved understanding of early mysticete evolution [1–8]. The extinct clades †Aetiocetidae and †Eomysticetidae help to understand transitional stages from toothed to baleen-bearing in mysticete evolution. Aetiocetids had fully erupted teeth, perhaps associated with proto-baleen [5,6,9], while eomysticetids had well-developed baleen, sometimes with remnant teeth [1]. But, the Oligocene record is not yet dense enough to show clear ancestor–descendant successions in species with teeth only, teeth-and-baleen and baleen-only [10]. Further, the rostrum—typically with thin bones and open sutures—often does not preserve well except in concretionary specimens which, in turn, may be of demanding preparation.

Here we describe a new genus and species of Mysticeti from the lower part of the Kokoamu Greensand, in the Canterbury Basin of southern New Zealand. Foraminiferal dating, elaborated below, places the whale in the uppermost Whaingaroan local stage, about 27.5 Ma, making the whale one of the oldest described baleen whales other than Eomysticetidae and other more stem-ward mysticetes. The source horizon is about 2 m above the intensely bioturbated globally widespread ‘middle’ Oligocene unconformity, in turn, close to the Rupelian–Chattian boundary. Baleen is inferred by the presence of a skeletal correlate on the maxilla: the presumed sulcus for the superior alveolar artery. The specimen has well-preserved tympanoperiotic bones which, in Cetacea, provide useful phylogenetic signal and diagnostic power [7,11–14].

## 2. Material and methods

Anatomical terminology follows Mead & Fordyce [15], modified and/or supplemented by Fordyce and co-workers [1,7,13], especially for the mysticete periotic and tympanic bulla. Here, the purpose of our cladistic analysis is to plot the position of a new archaic mysticete, OU 21981, rather than to resolve details of mysticete phylogeny. Thus, we added OU 21981 into the Tsai & Fordyce [13] matrix, including 91 taxa (one archaeocete as an outgroup, three odontocetes and 87 mysticetes) and 272 morphological characters (originally derived from Marx & Fordyce [16]). The original codings were retained to facilitate comparison with published phylogenies. In total, 105 characters were coded for OU 21981, representing 38.6% of 272 characters. Details of morphological coding for OU 21981 are as follows (?=missing or inapplicable):

???????0????0???????????????????????????11?00012120?????0??00????010010????0??100??0??????0????????????????????????????????????????0??1??01????01101001010100201020000000000000000000??000??00000011??0000000110111?0?1???3?????0?????1???????0?00??????????1000????????????????

Cladistic analyses were performed with heuristic analyses under the traditional search in ‘Tree analysis using New Technology’ (TNT v. 1.1 [17]). Settings for analyses are 10 000 maximum trees in the Memory option; 10 000 random stepwise-addition replicates; tree bisection reconnection branch swapping and saving 10 trees per replicate. Likewise, we present two different phylogenies that result from different settings (equal and implied weights, *k* = 3) to optimize comparisons with results of Tsai & Fordyce [13]. Because we do not aim to review the wider crown Mysticeti, we only show simplified phylogenies in the paper, but the detailed phylogenies with all the species and the full dataset for cladistics are included in the electronic supplementary material.

*Institutional abbreviation*. OU, Geology Museum, University of Otago, New Zealand.

## 3. Systematic palaeontology

Cetacea Brisson, 1762

Mysticeti Gray, 1864

Incertae familiae

*Toipahautea waitaki* gen. et. sp. nov.

(Figures 2–10)

*LSID.* urn:lsid:zoobank.org:pub:58C16BC5-B7D9-446D-B261-378F3A70ED73

*Holotype. †Toipahautea waitaki* is known only from the holotype OU 21981: a disarticulated partial skull (parts of the maxillae and premaxillae, left nasal, frontals, squamosals, exoccipitals, basioccipital and supraoccipital), incomplete mandibles, left tympanic bulla and periotic, hyoid(?), atlas, axis, two thoracic vertebrae, two scapulae, a partial humerus, two radii and ribs. Most of the elements were disarticulated but associated when excavated.

**Figure 1.**
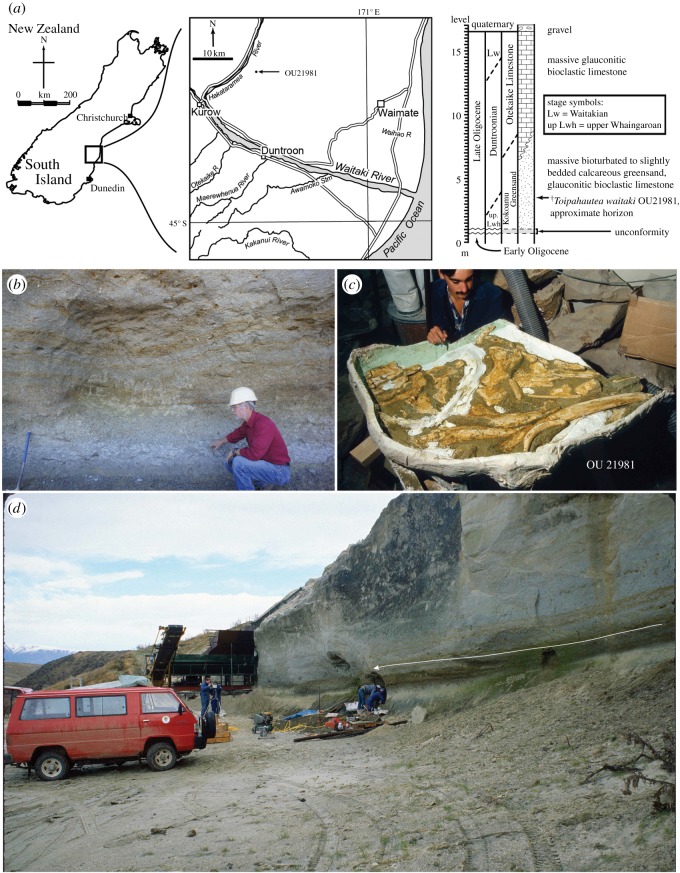
Geological map, excavation site and initial preparation of ^†^*Toipahautea waitaki* OU 21981. (*a*) General map and geological horizon of ^†^*Toipahautea waitaki* OU 21981; (*b*) excavation site of ^†^*Toipahautea waitaki* OU 21981 with RE Fordyce as a scale bar (photo ©CH Tsai); (*c*) initial preparation of ^†^*Toipahautea waitaki* OU 21981 by A. Grebneff (photo ©RE Fordyce); (*d*) scene of excavating ^†^*Toipahautea waitaki* OU 21981 (photo ©RE Fordyce); the arrow marks the level of the typical basal Duntroonian brachiopod shellbed.

*Preservation, size and ontogenetic age.* The skull was preserved ventral-up; figure 1*c* shows preparation of the underside, which is dominated by dorsal faces of many bones. The right mandible was medial (lingual)-up, while the left mandible was obliquely dorsomedial; the exposed surfaces in both show polished erosion, presumably bioerosion.

The occipital condyles preserve an almost complete outline (width: 92.3 mm), though the margins are eroded; outlines suffice to compare with those of ^†^*Horopeta umarere* (143.8 mm, estimated skull length: 1.5–1.6 m) to estimate the skull length of OU 21981. Simple scaling suggests that the condylobasal length of OU 21981 is about 1 m. In turn, based on simple scaling between ^†^*Horopeta umarere* and OU 21981, OU 21981 had a body about 5 m long, smaller than ^†^*Horopeta umarere* (6.5–7.5 m).

The open basioccipital/basisphenoid synchondrosis suggests that OU 21981 is a physically immature individual, either juvenile or sub-adult age class, based on the ontogenetic sequence of ossification in extant balaenopteroids [18]. Some posterior rostral elements are articulated, suggesting firm or closed sutures, although other sutures are open (e.g. exoccipital–squamosal); again, this is consistent with physical immaturity. On the atlas, the condyloid facets (articular surfaces) of the occipital condyles are smooth, without the pitting seen in juvenile mysticetes. In spite of the vagaries of preservation, and some possibly immature morphology, we show below that OU 21981 is sufficiently informative to establish a new species with emphasis on distinct and diagnostic tympanoperiotic morphology.

*Locality and horizon.* OU 21981 (field number REF-28.1.88.2) was recovered from the Hakataramea Valley, South Canterbury, South Island, New Zealand (figure 1). It was collected by R.E.F., A. Grebneff, and C.M. Jones on 28–30 January 1988 from an active quarry, known informally as Haughs' Quarry; at time of collecting, the quarry was owned by Foveran Station and operated by Waitaki Transport Limited. The latitude and longitude are 44^o^39′41′′ S and 170^o^39′00′′ E; the New Zealand Fossil Record number is I40/f0173 (http://www.fred.org.nz/), with the associated grid reference of I40: 2323565 5613665. The type location is about 20 m east of the site of ^†^*Horopeta umarere* and 2 m lower in the section.

On stratigraphy, OU 21981 (I40/f0173) is from massive bioturbated to diffuse-bedded limonitic calcareous greensand with sparse macroinvertebrates (mainly *Lentipecten* scallops and terebratulid brachiopods) and abundant foraminifera. The horizon is the lower part of the incompletely exposed Kokoamu Greensand, 1–1.3 m below a diffuse brachiopod–pectinid rich shellbed (I40/f0175) that is also conspicuous elsewhere in the southern Canterbury Basin. The brachiopod–pectinid rich shellbed generally occurs low in the Kokoamu Greensand, and has long been taken to approximate the base of the Duntroonian Stage.

R.E.F. provided matrix from the OU 21981 (I40/f0173) whale horizon, and adjacent higher levels (I40/f0174, and the brachiopod–pectinid rich shellbed at I40/f0175), to N. de B. Hornibrook for studies on the biostratigraphy of the benthic foraminiferan *Notorotalia*. Hornibrook [19] identified I40/f0174, which overlies the OU 21981 whale horizon, as the lowest occurrence of the Duntroonian index foraminiferan *N. spinosa*. Accordingly, I40/f0174 is the base of the Duntroonian stage, dated at 27.3 Ma and low in the Chattian (correlations from Raine *et al*. [20]).

Foraminifera from the OU 21981 (I40/f0173) whale horizon include *Notorotalia* cf. *N. stachei macrostachei* (R.E.F. observations), identified by the lack of regular dorsal ornament of *N. spinosa*: the latter is seen in the overlying I40/f0174. Thus, OU 21981 is upper Whaingaroan in age, more than 27.3 Ma and for convenience rounded to 27.5 Ma. See Tsai & Fordyce [7] for further details of geological settings.

*Etymology.* Toi means origin and pahautea refers to whalebone/baleen in Maori, alluding to the origin of the early toothless and baleen-bearing mysticetes. Waitaki is a name for the wider region, including the Waitaki River (wai, water or river; taki, tears) into which the smaller Hakataramea River drains.

*Diagnosis. †Toipahautea waitaki* is interpreted as a chaeomysticete based on the presence of ‘baleen’ sulci on the ventral surface of the maxilla and lack of mandibular alveoli. ^†^*Toipahautea waitaki* has a unique combination of: massive size of periotic; well-developed superior process of the periotic; prominent elongation of dorsomedial margin of the internal acoustic meatus; prominent fissure between the fenestra rotunda and the aperture for the cochlear aqueduct; small medial posterior sulcus; the presence of the anteroexternal foramen; the presence of the sigmoidal cavity; the presence of the elliptical foramen; horizontal sigmoidal cleft far anterior than the anterior margin of the sigmoidal process; posteromedial margin of the bulla orienting slightly anteromedially.

^†^*Toipahautea waitaki* differs from Oligocene-toothed mysticetes (unranked ^†^*Coronodon havensteini*, †Aetiocetidae and near-coeval/near-sympatric †Mammalodontidae) in greater condylobasal length; maxillae dorsoventrally thin and inferred to be long and laterally shelving; lack of functional teeth; rectangular supraorbital process of the frontal with mediolateral width greater than anteroposterior length; postorbital process of the frontal less developed; paroccipital process of the exoccipital extends posteriorly beyond the level of the occipital condyles; laterally deflected coronoid process; larger periotic and bulla; well-developed superior process; dorsal elongation of posterodorsal margin of the internal acoustic meatus; the presence of the anteroexternal foramen.

^†^*Toipahautea waitaki* differs from near-coeval/near-sympatric baleen-bearing †Eomysticetidae in lack of secondary squamosal fossa; robust supraorbital process with prominent pre- and postorbital processes; paroccipital process of the exoccipital extends posteriorly beyond the level of the occipital condyles; larger and more inflated periotic; well-developed superior process; dorsal elongation of posterodorsal margin of the internal acoustic meatus; tabular posterior process of the periotic slightly oriented ventrally; rounded-oblique posterior face on tympanic bulla, rather than a bilobed profile with interprominential notch; triangular and laterally deflected coronoid process.

^†^*Toipahautea waitaki* differs from Oligocene ^†^*Sitsqwayk* in a rectangular and transversely elongated supraorbital process of frontal; the profile of tympanic bulla more rounded instead of dorsoventrally compressed in medial view; the absence of distinct interprominential notch.

^†^*Toipahautea waitaki* differs from near-coeval/near-sympatric ^†^*Horopeta umarere* in laterally deflected coronoid process of the mandible; larger pars cochlearis; dorsal elongation of posterodorsal margin of the internal acoustic meatus; better-defined mallear fossa; more deeply excavated median promontorial groove.

^†^*Toipahautea waitaki* differs from near-coeval/near-sympatric ^†^*Whakakai waipata* in triangular anterior process of the periotic in medial view; small medial posterior sulcus; cylindrical and dorsoventrally narrow posterior process of the periotic.

^†^*Toipahautea waitaki* OU 21981 differs from all more crown-ward baleen-bearing mysticetes (Balaenopteridae, Cetotheriidae/Neobalaenidae, Balaenidae, Eschrichtiidae, ^†^*Parietobalaena*, and ^†^*Diorocetus* and others) in tympanoperiotic features including: unfused posterior processes of the periotic and tympanic bulla; the presence of a fovea epitubaria for the accessory ossicle on the ventral margin of the anterior process; a well-developed superior process; the presence of a sigmoidal cavity; the presence of an elliptical foramen; marked horizontal inflection on the sigmoid cleft of the sigmoid process.

## 4. Description/results

**Figure 2.**
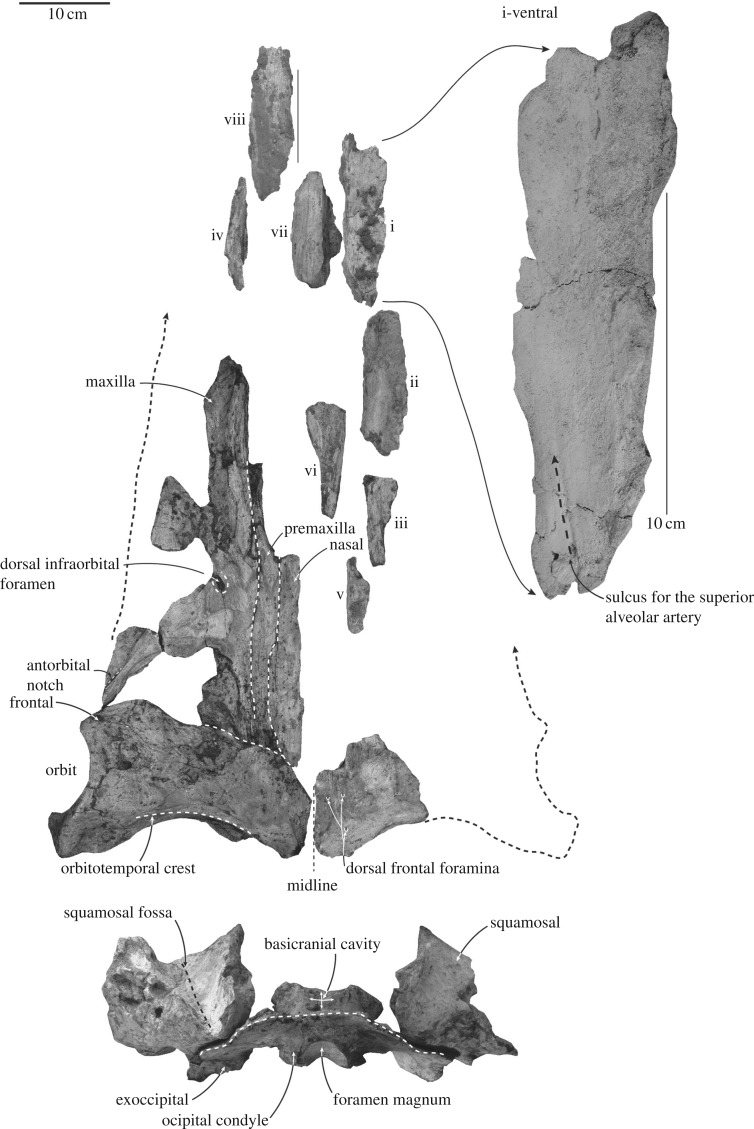
Layout of preserved skull and mandible materials of ^†^*Toipahautea waitaki* OU 21981. The enlarged diagram highlights a possible presence of sulcus for the superior alveolar artery on the ventral surface of maxilla; see text for detailed explanations of eight isolated pieces (i–viii).

**Figure 3.**
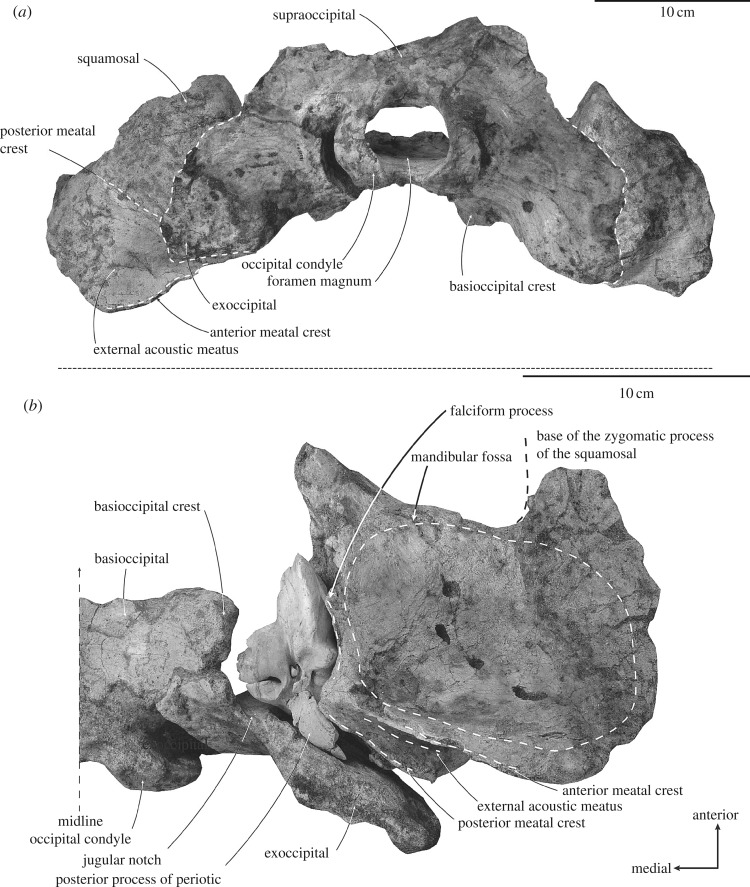
Occipitals and squamosals of ^†^*Toipahautea waitaki* OU 21981. (*a*) Posterior view of the occipitals and squamosals and (*b*) ventral view of the exoccipital and left squamosal with associated left periotic in place.

**Figure 4.**
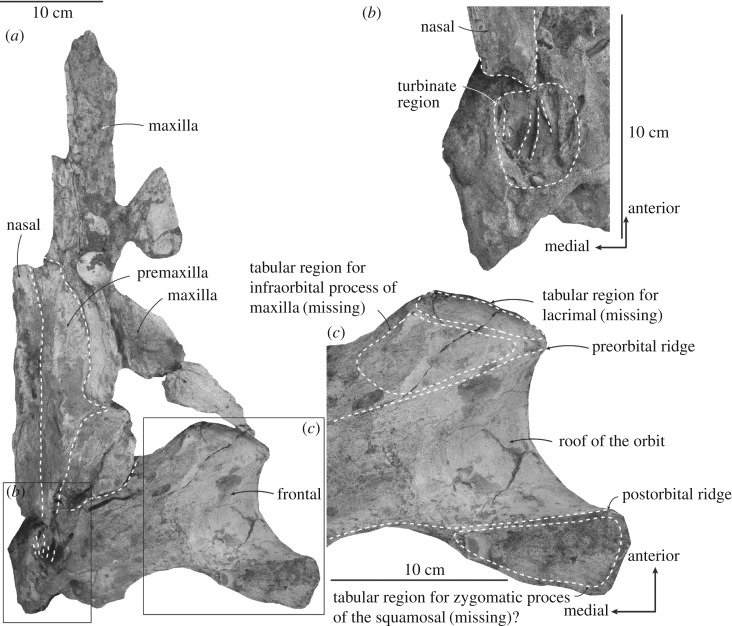
Left frontal, maxilla, premaxilla and nasal of ^†^*Toipahautea waitaki* OU 21981. (*a*) Ventral view of the left frontal, maxilla, premaxilla and nasal; (*b*) detailed view of the turbinates; and (*c*) details of the frontal in ventral view.

*Skull* (figures 2–4; table 1). The left frontal is slightly displaced and cemented by limonitic matrix to adjacent bones. The relative position of preserved skull parts as arranged in figure 2 is based on a referred specimen of *Mauicetus parki* (OU 22545); the left frontal complex, including nasal, premaxilla and maxilla, is oriented based on the assumption that main anteroposterior axis of nasal is parallel with the main axis of the skull. In addition, the right frontal preserves the midline suture; when reflected, the right frontal closely matches the orientation of the left frontal presented in figure 2 (the left frontal does not preserve the midline suture). There are some disarticulated bones, which are noted below if the associated features are revealing. Measurements are listed in table 1.

**Table 1.** Measurements (millimetres, ±0.5 mm) of the skull and mandible of ^†^*Toipahautea waitaki* OU 21981.

greatest preserved length of the skull, from postorbital process to the anterior tip of the preserved maxilla (not including the disarticulated occipital complex)	553.8
length of the orbit	100.2
dorsoventral depth of the orbit	37.9
greatest preserved length of the nasal	244.8
width of occipital condyles	92.3
width of foramen magnum	52.9
height of foramen magnum	35.5
greatest preserved length of the mandible (right)	840
greatest preserved height of the mandible, from coronoid process to ventral margin (broken)	195
greatest preserved height of the mandibular body (broken)	89
greatest preserved width of the mandible	45

### 4.1. Occipitals

The supraoccipital is largely missing, retaining the portion anterodorsal to the foramen magnum; the supraoccipital, basioccipital and exoccipitals are fused. The profile of the supraoccipital is uncertain, but based on the remaining part anterior to the foramen magnum, it probably rose forwards at about 45°. The exoccipitals are relatively complete, with small and laterally eroded occipital condyles and posterolaterally oriented paroccipital processes. The lateral and ventral margins of the occipital condyles are eroded (width of occipital condyles: 92.3 mm; compare atlas width, below), but consistent with short condyloid pedicles. Each condyle is roughly crescentic with a deep ventral intercondyloid notch. The foramen magnum is elliptical. Each exoccipital is eroded along the margins, thin and roughly vertical-oriented; the paroccipital process forms lateral flanges and orients posterolaterally so that the posteriormost point of the paroccipital process is far posterior to the condyles. There is no clear trace of stylohyal articulation or fossa for a posterior sinus which, as indicated by an open elliptical foramen, was presumably present [21].

The right dorsal condyloid fossa is more developed than the left side; the depression is about thumb-size and descends lateroventrally from the level of the dorsal margin of the foramen magnum to a point the half of the dorsoventral height of the foramen magnum. The jugular notch is broadly rounded, wide, ventrolateral to the occipital condyles in posterior view and passes up into the braincase via a broad shallow groove without a visible hypoglossal foramen. The basioccipital forms a semicircular arcade in posterior view, with the crests curving down laterally. In ventral view, each basioccipital crest is eroded and bulbous, with uncertain orientation; there is no obvious contribution of exoccipital. Anteriorly, the surface of the basioccipital is eroded; judging from the anterior surface of the basioccipital and a partial disarticulated basisphenoid, the basioccipital/basisphenoid synchondrosis is open. The right basisphenoid contains the presumed dorsal carotid foramen, which is ovoid to slit-like, 15.5 mm in diameter and oriented dorsomedially.

### 4.2. Squamosal

The squamosals retain the squamosal fossa, mandibular fossa, periotic fossa and posterior (meatal) margins, but each lacks the zygomatic process. Both squamosals can be roughly articulated close to the exoccipital within a few millimetres, with the periotic in place; when both squamosals are articulated with the exoccipital, the width from the lateral margin of left squamosal to the midline (centre of foramen magnum) is about 235 mm, making the estimated width across both squamosals 470 mm. In dorsal view, the squamosal fossa descends anteroventrally approximately at 45° (preserved anteroposterior length: 119 mm point–point). Ventrally, the mandibular fossa is roughly rectangular to circular, moderately concave and gently eroded in front of the anterior meatal crest; the left mandibular fossa is approximately 86 mm long by 108 mm wide. The ventral margin of the anterior meatal crest is eroded; thus, the shape of postglenoid process is uncertain, but it descends approximately 45° posteroventrally. The external acoustic meatus is dorsoventrally shallow proximally and expands dorsoventrally distally, forming a triangular surface in posterior view (figure 3*a*); the distal part of the posterior meatal crest abruptly (approx. 60°) ascends dorsolaterally at about 50 mm away from the periotic fossa. The posterior process of the bulla is missing, and the few long and shallow depressions on the posterior surface of the external acoustic meatus do not clearly indicate the size, contacts and lateral extent of the posterior process of the bulla. The spiny process is not well developed, but marks a gentle, rounded depression. The falciform process, anterior to the spiny process, is shelf-like, semicircular and on the median edge of the mandibular fossa. The periotic fossa is largely rounded and gently concave.

### 4.3. Frontal

The left frontal is well preserved dorsally, rectangular to slightly dumb-bell shaped in dorsal view; the right frontal is partly preserved (figure 2). Sutures with adjacent bones are eroded by a few millimetres, so that contacts are uncertain. Anteriorly, the straight anterolateral margin of the supraorbital process, close to the antorbital notch of the maxilla, orients posterolaterally; the anteromedial margin of the supraorbital process is eroded and slightly concave posterior to the maxilla. Three possible dorsal frontal foramina are present on the right frontal (the left frontal shows no foramina, possibly because of erosion) and open dorsally to anterodorsally.

The posterior margin of the supraorbital process is concave in dorsal view, with a shallow orbitotemporal crest along the posterodorsal margin and a postorbital ridge—eroded laterally—below. The two ridges delimit a shallow, wide fossa for the m temporalis on the posterior face of the frontal, partly visible ventrally.

The lateral margin of the orbit is concave, viewed dorsally, with the eroded postorbital process more lateral than the preorbital process; the postorbital process is prolonged posterolaterally. Orbital length is about 100.0 mm and dorsoventral depth 38.0 mm. In ventral view, the roof of the orbit between the preorbital and postorbital ridges forms a concave, subconical surface with little erosion. Two foramina are presumably for the frontal diploic vein.

Other ventral features are damaged. The triangular and slightly convex surface on the postorbital process is eroded, without any distinct facet to oppose the tip of the zygomatic process of the squamosal; the postorbital ridge is damaged here, but is present medially. Anteriorly, two somewhat polished eroded tabular surfaces of frontal indicate the suture for the lacrimal, and the surface facing the infraorbital process of the maxilla (figure 4*a,c*). The tabular region for the missing lacrimal is long, rectangular and oriented ventromedially, consistent with a loosely sutured thin plate-like lacrimal. The tabular surface opposing the infraorbital process of the maxilla (missing) is trapezoid and oriented ventrally. The preorbital ridge is eroded laterally, but developed medially, where it carries a subcircular sulcus, probably from the ethmoid foramen (by analogy with *Balaenoptera bonaerensis*). The orbitosphenoid is not visible.

Ventrally, the turbinate region (figure 4*a,b*) is broadly subspherical and oriented slightly medioventrally; the dimension is about 45 × 32 mm and up to 20 mm deep. The turbinate region includes three prominent and up to 13 (broken) nasal turbinates on the ventromedial margin, and marks the presence of ethmoid labyrinth or olfactory recess; the turbinates are thin and fine ridges. There is no trace of a paranasal sinus lateral to the turbinates.

### 4.4. Nasal

The left nasal (figures 2 and 4*a*) is long, narrow and rectangular (preserved length: 244.8 mm; width: 34 mm), and dorsoventrally flattened; the dorsal surface is flat while the eroded ventral surface shows a long and gently concave groove along the entire nasal. The anterior margin is eroded, but judging from the curvature of the premaxilla lateral to the nasal, the preserved nasal includes the anterior end at the posterior margin of the narial fossa. Posteriorly, the margin is eroded and profile uncertain, but at least it reaches to the level of anteroposterior midline of the frontal. The lateral face of the nasal is slightly overlain by the premaxilla on the ventral surface, but not on the dorsal surface.

### 4.5. Rostrum

The posterior portion of the maxilla indicates a broad and thin rostrum, as noted during the field excavation and widely seen in other baleen-bearing Plicogulae. Eight disarticulated pieces are provisionally identified as rostral; their distinctive form rules out other parts of the skull or skeleton (figure 2). One thin piece of right maxilla (figure 2(i)) preserves a sulcus ventrally, interpreted as the sulcus for the superior alveolar artery (*sensu* [22]). The sulcus is long (preserved length: 41 mm; width: 4 mm), shallow, slightly widening and then disappearing anteriorly; the orientation is uncertain, but is likely slightly anteromedially as seen in some extant Plicogulae. Of three other pieces of presumed maxilla in figure 2, (ii) and (iii) belong to the right while (iv) belongs to the left. In all, the medial margins are grooved and dorsoventrally thickened medially, consistent with a mobile suture for the premaxilla. The cross sections of figure 2(pieces i and ii) are triangular, thick medially and thin laterally, consistent with the broad and thin rostrum as seen in extant Plicogulae.

Four pieces of presumed premaxilla (figure 2(v–vii)) are from the right, whereas figure 2(viii) belongs to the left. The medial margin of figure 2(piece v) has a flat and smooth vertical surface, suggesting attachment with the nasal. The medial margin of figure 2(piece vi) is twisted from posteriorly (facing medially) to anteriorly (facing dorsomedially), suggesting a position at the nares, anterior to the anterior tip of the nasal. The dorsal margins of figure 2(pieces vii and viii) show broad and flat surfaces, suggesting anterior broadening parts of the premaxilla.

The left facial complex includes nasal, premaxilla and maxilla. Some details are obscured by limonitic matrix. Here, the slightly eroded posterior margins of both maxilla and premaxilla are slightly anterior to the posterior margin of the nasal. The gentle outwards curvature of the premaxilla, anterior to the nasal, marks the external narial fossa as seen in dorsal view. The ascending process of the premaxilla, posterior to the narial fossa, is long, slender and rectangular. The posterior margin of the maxilla, though damaged, indicates a short and triangular ascending process of the maxilla. One large sub-oval dorsal infraorbital foramen opens slightly posterior to the external narial fossa.

The incomplete antorbital notch on the maxilla has a broad groove, presumably for the facial nerve, oriented posterolaterally towards the anterolateral margin of the supraorbital process of the frontal. Here the lateral margin of the antorbital process is slightly eroded; the original margin external to the antorbital process of the maxilla below the antorbital notch is thin.

### 4.6. Mandible

**Figure 5.**
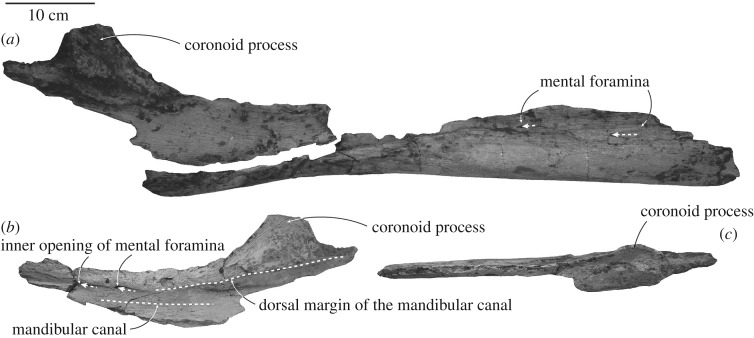
Right mandible of ^†^*Toipahautea waitaki* OU 21981. (*a*) Lateral view of the two main pieces of the right mandible; (*b*) medial view of the right mandible with coronoid process; and (*c*) dorsal view of the right mandible with coronoid process.

Both mandibles lack surface detail dorsomedially, presumably the result of bioturbation when exposed on the sea floor; lateral faces are better-preserved (figure 5; table 1). The anterior symphyseal surface, mandibular condyle, angular process, panbone and mandibular foramen are lost. The better-preserved right mandible has two complementary but not closely adjoining pieces: the body (710 mm long) and the region of the coronoid process (377 mm long). Each mandible is slightly laterally and dorsally bowed, interpreted as natural curvature rather than postmortem distortion. The cross section is compressed oval anteriorly, but with the ventral part more rounded and dorsal compressed in mid-length, giving an inverted comma-shaped cross section. A chunk of the apical lateral face of the left symphyseal region has a large, forward-facing, dorsal foramen, but whether a mental foramen or an alveolus is uncertain. The dorsal crest is preserved anteriorly for about 150 mm on the right mandible, with no large alveoli as seen in some eomysticetids, no alveolar groove and no gingival foramina. The mental foramina are long, deep and oval, opening forward into sulci (figure 5*b*). On the right, 100 mm of coronoid crest is preserved before about 37 mm of broken margin for the possibly triangular coronoid process; the gradually sloping coronoid crest, and steeper posterior margin, suggest that little is lost from the coronoid process. The medial wall of the right mandibular canal is deep and broadly concave, but its overall profile is uncertain. The dorsal margin of the mandibular fossa, ventral to the coronoid process, is thick and robust. Laterally and more ventrally, bone thins to less than 3 mm towards the now-lost panbone. Ventrally, the rounded profile lacks an insertion for gular muscles or the mylohyoid groove. Medially, the right mandible shows a fine groove for Meckel's cartilage, running 280 mm from mid-depth anteriorly to the ventral surface posteriorly.

### 4.7. Periotic, left

**Figure 6.**
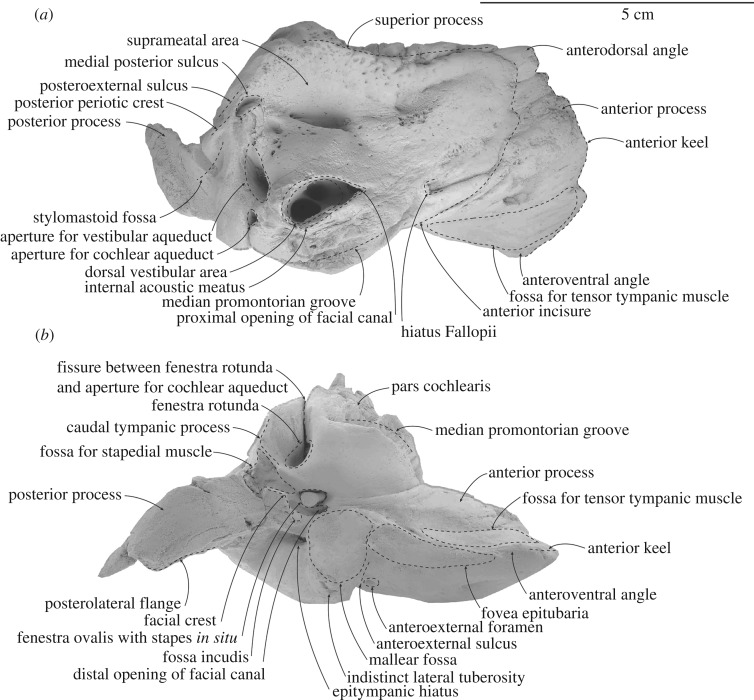
Details and interpretations of left periotic of ^†^*Toipahautea waitaki* OU 21981. (*a*) Dorsal view and (*b*) ventral view.

**Figure 7.**
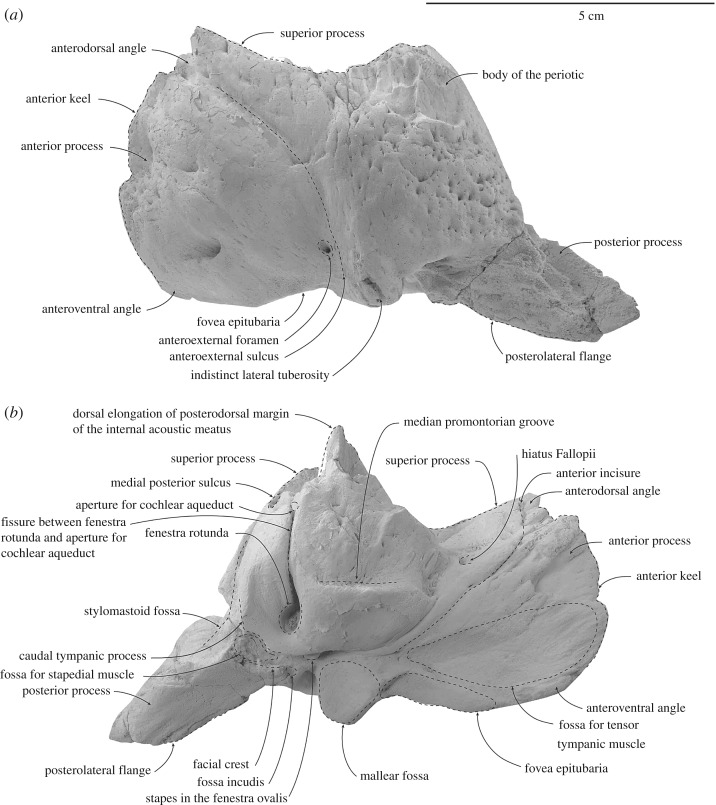
Details and interpretations of left periotic of ^†^*Toipahautea waitaki* OU 21981. (*a*) Lateral view and (*b*) medial view.

When the periotic is articulated in the periotic fossa, the posterior process of the periotic is oriented posterolaterally and terminates about 25 mm away from the periotic fossa, far away from the lateral surface of the skull; thus, the skull is amastoid (figures 6 and 7; table 2). The lateral tuberosity is not well developed. When the periotic sits against the periotic fossa, the space between the anteroexternal sulcus of the periotic and the periotic fossa presumably marks the path of middle meningeal artery; the lateral tuberosity is not protruding beyond the level of the falciform process. The anterior process is broadly triangular in medial view and tapering towards the anterior keel in dorsal view. The anterior keel is slightly convex anteriorly and runs vertically from the anterodorsal to the anteroventral angle. The medial surface of the anterior process is irregularly fissured and perforated, while the lateral surface is smooth to finely perforated, with a prominent foramen or pit roughly at the level of the anteroventral angle. The anteroexternal sulcus is a shallow groove, curving anteriorly from the lateral tuberosity up to the anterodorsal angle. One anteroexternal foramen is present anterodorsal to the lateral tuberosity. The lateral tuberosity is poorly developed, blunt and bulbous; when articulated, it does not project into the adjacent squamosal.

**Table 2.** Measurements (millimetres, ±0.5 mm) of the periotic of ^†^*Toipahautea waitaki* OU 21981.

maximum diameter of the aperture for the cochlear aqueduct	2.6
maximum diameter of the aperture for the vestibular aqueduct	10.1
maximum diameter of the internal acoustic meatus	15.1
maximum diameter of the proximal opening of the facial canal	3.6
maximum diameter of the dorsal vestibular area	11.5
maximum diameter of the fenestra rotunda	6.9
maximum diameter of the fenestra ovalis	5.4
maximum diameter of mallear fossa	11.3
maximum diameter of distal opening of the facial canal	1.9
maximum diameter of the fovea epitubaria for accessory ossicle	13.9
length of anterior keel, from tip of anterodorsal angle to tip of anteroventral angle	45.7
length from anteroventral angle to tip of lateral tuberosity	37.7
length from anteroventral angle to anterior margin of the fovea epitubaria	16.3
maximum diameter from anteroventral angle to posterior margin around the aperture for the cochlear aqueduct of pars cochlearis	54.1
length of anterior process, from anteroventral angle to anterior incisure	26.3
length of pars cochlearis, from anterior incisure to posterior margin around the aperture for the cochlear aqueduct of pars cochlearis	27.8

The fovea epitubaria is long with a marked depression posteriorly; there is no pedicle plate and/or accessory ossicle attached. The ventral margin of the anterior process is slightly ventral to the level of the ventral margin of the pars cochlearis. The mallear fossa is concave with well-defined margins. The inner margin of the mallear fossa overhangs the facial sulcus and a fissure originating at the distal opening of the facial canal, passing posteroventrally to the facial crest. A gentle depression, lateral to the start of facial crest and posterior to the mallear fossa, may be the fossa incudis. Adjacent, the epitympanic hiatus for receiving the spiny process is dorsal to the posterolateral flange.

The distal opening of the facial canal opens roughly at the same level as the anterior margin of the fenestra ovalis, with the facial sulcus separated from the fenestra ovalis by a narrow crest. The fenestra ovalis is slightly elliptical, with a long anteroposterior diameter. Posterior to the fenestra ovalis, the facial sulcus runs posteroventrally onto the base of the posterior process of the periotic; the posterior process of the tympanic bulla was not fused to the periotic and is missing. The pitted fossa for the stapedial muscle, posterior to the fenestra ovalis and onto the base of the posterior process, is slightly irregular to hemispherical and large. The facial crest forms part of the ventral surface of the fossa for the stapedial muscle; the medial edge of the facial crest is slightly eroded and damaged.

The anterior surface of the pars cochlearis gradually grades onto the medial surface of the anterior process. The anterior incisure, which marks the anterior limit of the pars cochlearis, slightly curves up to the anterodorsal angle. A small and rectangular foramen, posterior to the anterior incisure and at the level of the aperture for the cochlear aqueduct, may be the hiatus Fallopii. The posterodorsal margin of the internal acoustic meatus is slightly elongated dorsally. The proximal opening of the facial canal, the dorsal vestibular area and the aperture for the cochlear aqueduct are roughly in line. The proximal opening of the facial canal is elliptical, the dorsal vestibular area slightly irregular to elliptical and the aperture for the cochlear aqueduct rounded; the transverse crest of the internal acoustic meatus is well below the dorsal margin of the internal acoustic meatus. The size of the dorsal vestibular area is more than twice of that of the proximal opening of the facial canal. The foramen singulare in the dorsal vestibular area is large, about half size of the dorsal vestibular area, and rounded to elliptical. The spiral cribriform tract and the area cribrosa media are distinct and both are rounded; the area cribrosa media is perforated ventrally.

The apertures for the vestibular aqueduct and cochlear aqueduct, and the fenestra rotunda, lie roughly in a transverse line. The aperture for the cochlear aqueduct on the posteromedial corner of the pars cochlearis is rounded and smaller than the aperture for the vestibular aqueduct. The aperture for the vestibular aqueduct is slit-like and oriented anteromedially. A long fissure connects the aperture for the cochlear aqueduct and the fenestra rotunda. Ventrally, the fenestra rotunda is irregular to rounded and slightly larger than the fenestra ovalis, and opens at the posteromedial corner of the pars cochlearis. The caudal tympanic process is transversely thin, extending slightly posteriorly and broken posteroventrally. The median promontorial groove, anterior to the fenestra rotunda, runs horizontally, reaches anteriorly to the level of the anterior margin of the pars cochlearis and is deeply excavated. The ventral surface of the pars cochlearis, anterior to the fenestra rotunda and medial to the fenestra ovalis, is rounded.

The junction of the body and pars cochlearis, as seen in dorsal view, is irregularly perforated and deeply excavated. The superior process is prominent and concave between the anterodorsal angle and dorsal edge of the posteroexternal sulcus; the superior process is slightly lower than the dorsal elongation of the posterodorsal margin of the pars cochlearis. The deep suprameatal fossa is bounded between the superior process laterally and dorsally elongated lateral margins of the internal acoustic meatus medially. The medial posterior sulcus, posterior to the suprameatal area and posterolateral to the aperture for the vestibular aqueduct, is small and lunate. The posterior periotic crest is robust, separating the medial posterior sulcus and the posteroexternal sulcus. The posteroexternal sulcus, posterior to the medial posterior sulcus, carries the posteroexternal foramen roughly at the level of one-third the dorsoventral height of the periotic; the posteroexternal sulcus runs down onto the lateral surface of the posterior process.

The posterior process is slightly cylindrical and becomes transversely thin distally; the height of the base of the posterior process is about one-third of the dorsoventral height of the periotic; the posterior process is oriented posterolaterally. The ventral surface for the posterior process of the periotic is smooth proximally and slightly ridged distally. On the posterior surface of the pars cochlearis, the stylomastoid fossa is deeply excavated on the lateral half, and forms a prominent depression on the dorsomedial surface of the posterior process.

### 4.8. Tympanic bulla

**Figure 8.**
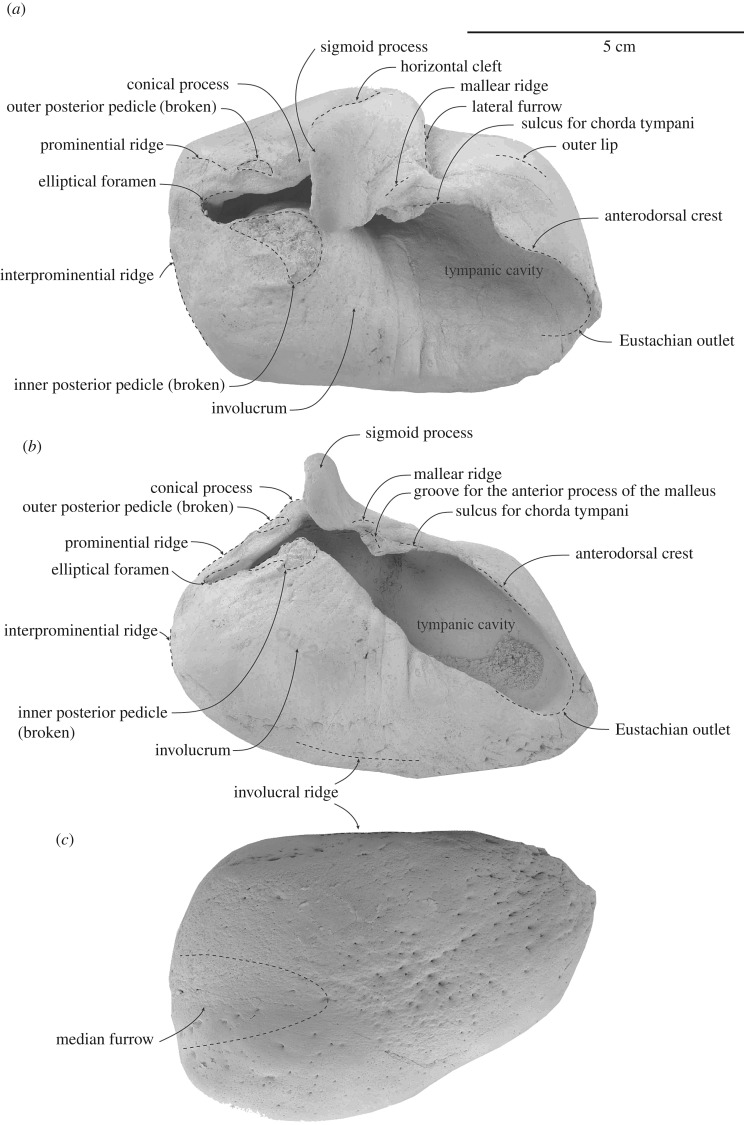
Details and interpretations of left tympanic bulla of ^†^*Toipahautea waitaki* OU 21981. (*a*) Dorsal view; (*b*) medial view; and (*c*) ventral view.

**Figure 9.**
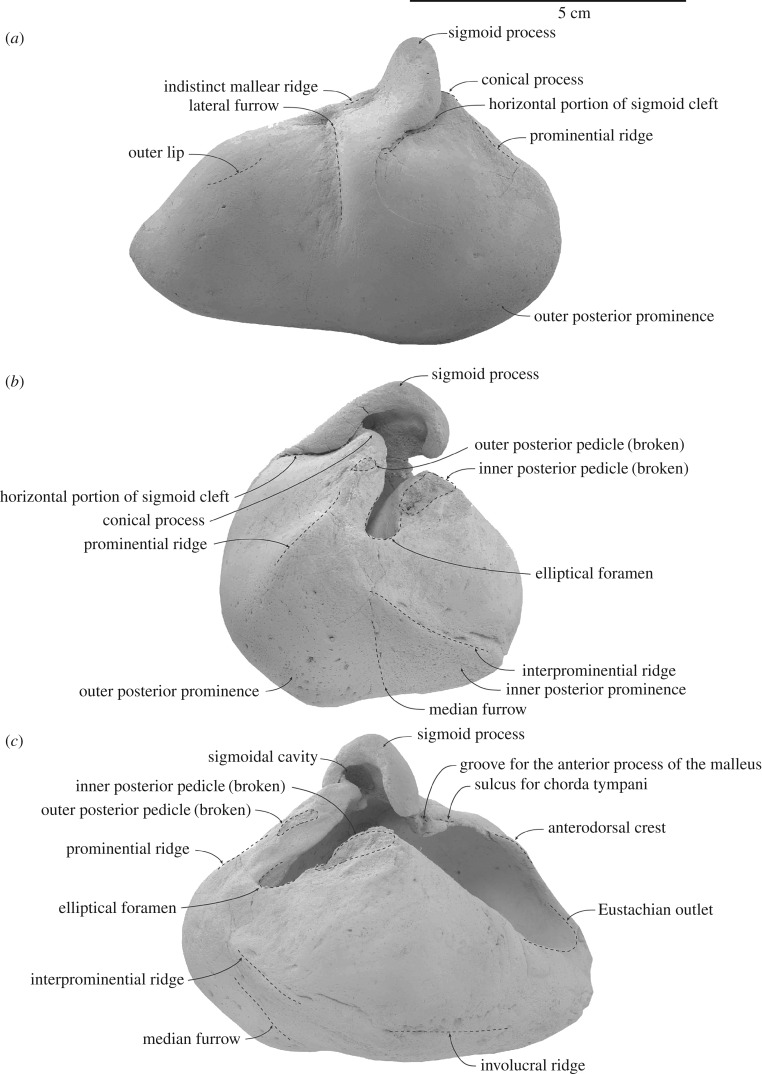
Details and interpretations of left tympanic bulla of ^†^*Toipahautea waitaki* OU 21981. (*a*) Lateral view; (*b*) posterior view; and (*c*) posteromedial view.

The posterior process of the left bulla is missing (figures 8 and 9; table 3). Its general profile is broadly rectangular in dorsal view with its anterior tip slightly protruded. The Eustachian outlet is directed anteromedially. There is no transverse ridge across the tympanic cavity. The smooth outer lip is slightly inflated anterolaterally, covering most of the tympanic cavity in dorsal view. The anterodorsal crest on the outer lip ascends smoothly from the apex of the bulla to the level of the dorsal origin of the lateral furrow. There is a step laterally from the anterodorsal crest to the sulcus for the chorda tympani. The sulcus for the chorda tympani lies along the dorsal edge of the outer lip and medial to the lateral furrow. A small ridge separates the latter sulcus from the remnants of the groove for the anterior process of the malleus. A lateral furrow is just anterior to the mid-length of the bulla. The mallear ridge, posterodorsal to the dorsal origin of the lateral furrow, is slightly bulbous and indistinct.

**Table 3.** Measurements (millimetres, ±0.5 mm) of the tympanic bulla of ^†^*Toipahautea waitaki* OU 21981.

maximum length, in dorsal view	77.4
thickness of involucrum across the anterior point of the base of inner posterior pedicle	29.6
maximum length of tympanic cavity, in dorsal view slightly turn medially	68.4
maximum width, in dorsal view	51.7
maximum height, from tip of sigmoid process to the ventral-most point in posterior view	55.9
height of involucrum, from the base of the anterior point of inner posterior pedicle to ventral point, perpendicular to the long axis of tympanic bulla	37
length of anterior lobe, from lateral furrow to anterior tip of tympanic bulla	36.9
length from tip of sigmoid process to anterior tip of tympanic bulla (from tip to tip)	64.8
length from tip of sigmoid process to anterior tip of tympanic bulla (from cross section to tip)	52.4
maximum length of posterior pedicle for posterior process on tympanic bulla at base	15.3

The sigmoid process, posterior to the mid-length of the bulla, projects dorsally and is roughly perpendicular to the main axis of the bulla; the dorsal margin is a rounded triangle in posterior view. The sigmoid cleft curves anteriorly slightly forward of the anterior face of the sigmoid process, curving more strongly (approx. 45°) and deeper than in ^†^*Horopeta* and ^†^*Whakakai*.

The sigmoidal cavity, on the posterior face of the sigmoid process, is broadly oval and marks the anterior limit of the tympanic sulcus. The tympanic sulcus arises on the posterior surface of the sigmoid process and runs along the base of the conical process towards the outer posterior pedicle. The conical process is gently elevated with a triangular apex (medial and lateral views, figures 8*b* and 9*a*); the anterior half and the apex of the conical process are roofed by the sigmoid process, and the medial margin of the conical process is roughly at the same level medially as the apex of the sigmoid process in posterior view.

The prominential ridge is indistinct and descends lateroventrally to the level of the interprominential ridge. The outer posterior pedicle, medial to the prominential ridge, is broken, and judging from the remaining base, it is thinner and more delicate than the inner posterior pedicle.

There is no interprominential notch (dorsal view, figure 8*a*), or distinctly projecting prominences, so that the posterior profile is oblique to rounded and not bilobed. The outer posterior prominence lies slightly more posterior than the inner posterior prominence. In posterior view, the outer and inner posterior prominences are of roughly equal size. The elliptical foramen is wide, deep, slightly oval and oriented ventrolaterally. The interprominential ridge is medioventrally oriented and reaches the posteromedial corner to merge with the involucral ridge medially. The median furrow is a broad and shallow groove, ventral to the interprominential ridge on the ventral surface of the bulla, and extends forward to the level of the conical process. The anterior half of the ventral surface of the bulla is pitted, smooth and broadly flattened.

The domed posterodorsal surface of the involucrum passes forward via a slight step on the dorsal surface to the flatter anterior surface. The broken base of the inner posterior pedicle sits posteriorly on the dome. The surface of the involucrum is smooth, with fine transverse creases on the posterior half. The involucral ridge is diffuse and faintly passes forward to the anteriormost point of the bulla; this ridge probably marks the insertion for the connective tissue along the basioccipital crest.

### 4.9. Atlas

**Figure 10.**
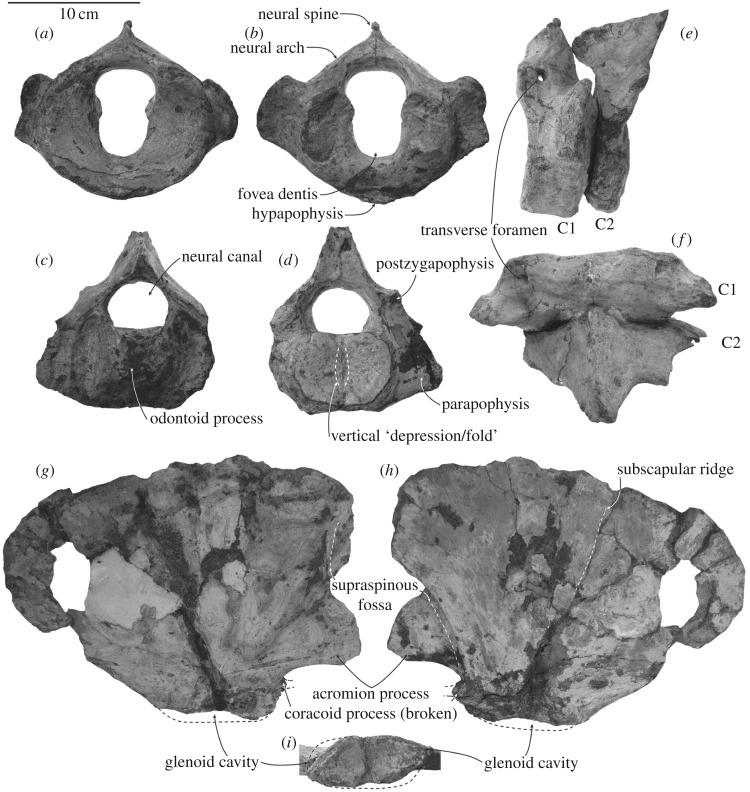
Postcranial elements of ^†^*Toipahautea waitaki* OU 21981. (*a*) Anterior view of the atlas; (*b*) posterior view of the atlas; (*c*) anterior view of the axis; (*d*) posterior view of the axis; (*e*) left lateral view of the atlas and axis in sequence; (*f*) dorsal view of the atlas and axis in sequence; (*g*) lateral view of the right scapula; (*h*) medial view of the right scapula; and (*i*) glenoid cavity of the left scapula.

The atlas is broadly rounded in anterior view, with an acutely pointed dorsal neural spine and deep transverse processes; the articular facets for the occipital condyles and the axis are smooth, not pitted as seen in young *Balaenoptera bonaerensis* [23] (figure 10; table 4). Each condyloid facet is roughly hemispherical and gently concave; ventrally the left and right condyloid facets are confluent. The base of each transverse process is high, robust, projecting dorsolaterally and about half the height of the body, roughly from the level of the transverse foramen to the ventral margin of the neural canal. The oval transverse foramen opens at the anterior half of the robust neural arch. The neural spine is short and roughly posterior to the anteroposterior midline of the neural arch. The anterior part of the neural spine of the axis articulates with the neural arch of the atlas just posterior to the neural spine of the atlas. The articular facets for the axis are slightly convex and broadly U-shaped, with a confluent ventral margin. The hypapophysis is indistinct at the posterior part of the ventral surface. The neural canal-fovea dentis (odontoid fovea) is approximately 8-shaped, separated by bilateral ridges for the transverse ligament. The fovea dentis is broadly U-shaped to rounded, and slightly smaller than the neural canal. The anterior width of the atlas across the eroded crests of condyloid facets (articular surfaces) is estimated as 118 mm, substantially wider than occipital condyle breadth of 93.0 mm.

**Table 4.** Measurements (millimetres, ± 0.5 mm) of the vertebrae of ^†^*Toipahautea waitaki* OU 21981.

	length of body	anterior height of body	anterior width of body
atlas (C1)	38	104.6	118.1
axis (C2)	37	93.2 (*broken*)	115.7 (broken)

### 4.10. Axis

The axis is broadly triangular in anterior view, with most of both transverse processes missing (figure 10). The odontoid process is broadly rounded, slightly elevated from the body. The margins of the vertically creased posterior epiphysis have a closed suture with the body. The base of the transverse process is transversely robust and well-developed dorsoventrally, more than half height of the body. Judging from the remaining base of the right transverse process, the transverse process projects posterolaterally. There is no indication of the vertebrarterial canal or perforating foramen on the transverse process. The neural arch is robust, with a strong overhanging postzygapophysis, on which the articular surface is roughly rectangular and facing almost ventrally. The robust neural spine projects anteriorly, almost level with the odontoid process, to articulate with the posterodorsal surface of the neural arch of the atlas. The posterior tip of the neural arch and the postzygapophysis both project beyond the posterior surface of the body. The dorsal margin of the neural arch descends gradually anteriorly. The neural canal is broadly rounded.

### 4.11. Ribs

Three relatively complete right ribs are of uncertain order and arrangement. Maximum preserved chord length is 293 mm. Two ribs preserve the proximal parts, showing some curvature; one is anteroposteriorly thin (10.4 mm), and presumably more anterior, while the other is thicker (21 mm), and more posterior.

### 4.12. Forelimb

Both forelimbs include the scapulae, and parts of both radii; the humerus and ulna are missing. Each scapula is broadly fan-shaped with an anteroposterior length longer than dorsoventral height and transversely thin (table 5). The robust base of the acromion process (right) projects slightly anteroventrally; the distal part is missing. The remnant base of the coracoid process is much smaller than the acromion process. The supraspinous fossa is broadly grooved along the anterolateral margin of the scapula down to the level of the ventral margin of the acromion process. The glenoid cavity is broadly oval and shallow. The subscapular ridge is broadly and slightly elevated on the medial surface. The anterior and posterior margins descend down to the glenoid cavity, roughly forming a right angle. The radius (not figured) is transversely flattened, broadly oval in cross section and slightly curving. The distal surface of the right radius is broken.

**Table 5.** Measurements (millimetres, ±0.5 mm) of the forelimb of ^†^*Toipahautea waitaki* OU 21981.

length of scapula (left)	297
height of scapula (left)	205
length of glenoid cavity	73
width of glenoid cavity	37
height of acromion process (right)	62
length of radius, as preserved	171

### 4.13. Phylogenetic placement of ^†^*Toipahautea waitaki*

**Figure 11.**
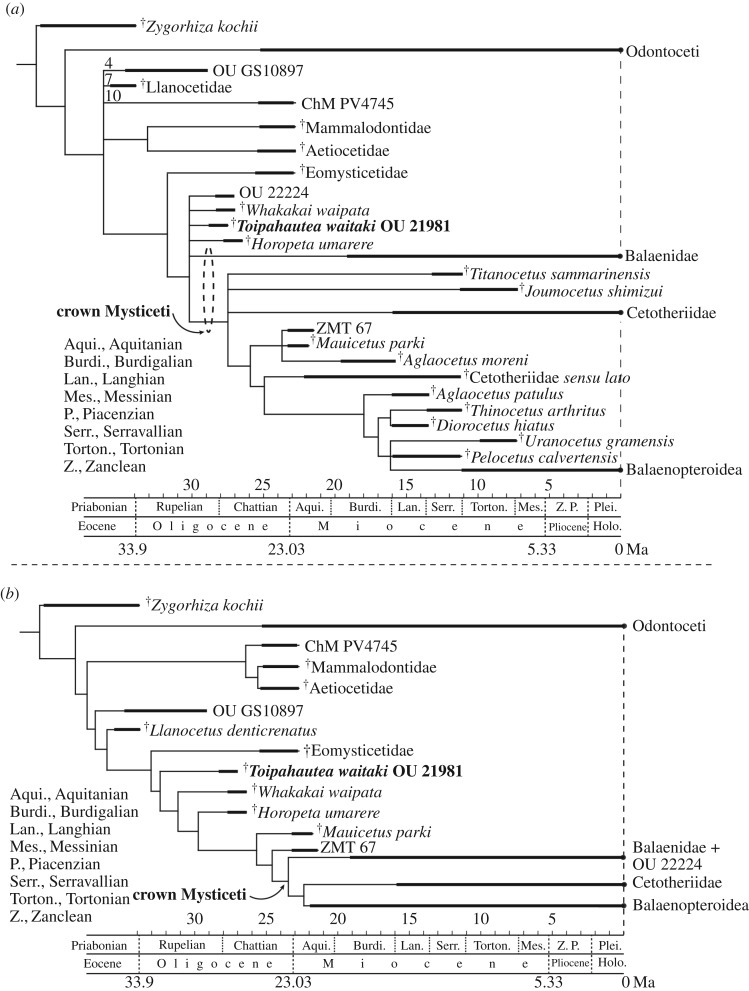
Simplified phylogenetic placements of ^†^*Toipahautea waitaki* OU 21981. (*a*) A strict consensus of 920 equally parsimonious trees (equal weights; tree length: 1283) and (*b*) a strict consensus of three most parsimonious trees (implied weights, *k* = 3; tree length: 117.438) (see electronic supplementary material, file for the full phylogenies).

An equal weights analysis produced 920 equally parsimonious trees with a tree length of 1283 (strict consensus tree: figure 11*a*). An implied weights analysis (*k* = 3) produced three equally parsimonious trees with a tree length of 117.438 (strict consensus tree: figure 11*b*). Full phylogenies with terminal taxa are in the electronic supplementary material. Under equal weights (figure 11*a*), ^†^*Toipahautea* is one branch of a polytomy that includes unresolved relationships amongst crown Mysticeti, ^†^*Horopeta*, ^†^*Whakakai* and undescribed OU 22224. In the implied weight analysis (figure 11*b*), ^†^*Toipahautea* is part of a ladder-like sequence between Eomysticetidae and crown Mysticeti; ^†^*Whakakai*, ^†^*Horopeta*, ^†^*Mauicetus* and undescribed ^†^*Mauicetus*-like ZMT 67 are also in this ladder-like sequence.

## 5. Discussion

### 5.1. ^†^*Toipahautea waitaki*: the oldest crown Mysticeti?

^†^*Toipahautea waitaki, †Whakakai waipata* and ^†^*Horopeta umarere* are all known from single specimens from the Kokoamu Greensand of the Waitaki region, and all are interpreted as Late Oligocene baleen-bearing archaic mysticetes. ^†^*Toipahautea waitaki* is the oldest, from low in the Greensand and in the upper Whaingaroan local stage (basal Chattian, about 27.5 Ma). ^†^*Whakakai waipata* is a little younger, from a distinctive brachiopod–pectinid rich shellbed at about the base of the Duntroonian Stage (no more than 27.3 Ma), while ^†^*Horopeta umarere* is higher, but far below the top of the Duntroonian (at 25.2 Ma). Further, three species form a succession at the same Hakataramea locality: ^†^*Toipahautea waitaki* (oldest), ^†^*Horopeta umarere* and ^†^*Mauicetus parki* (youngest, *ca* 24.7 Ma; see below). ^†^*Whakakai waipata* is from elsewhere, near Duntroon.

All the type-specimens of ^†^*T. waitaki*, ^†^*W. waipata* and ^†^*H. umarere* include character-rich tympanoperiotic complexes, in which phylogenetically revealing features are present early in ontogeny, supplementing the incomplete skulls. Phylogenetic analyses show the three species as more crown-ward than the clade †Eomysticetidae, but of uncertain relation to the crown Mysticeti, and included or excluded from the crown group depending on analytical settings [7,13].

According to the original definition of a crown group [24], the crown Mysticeti will include the most recent common ancestor (MRCA) of the six extant genera of baleen whales (*Balaena*, *Balaenoptera*, *Caperea*, *Eschrichtius*, *Eubalaena* and *Megaptera*) and all the descendants of the MRCA. †Eomysticetidae has consistently been identified as the sister clade to the crown Mysticeti, as in figure 11 (e.g. [2,3,16]), and is one of several stem mysticete clades, including: †Aetiocetidae, †Mammalodontidae, †Llanocetidae and some mysticetes from South Carolina, such as ^†^*Coronodon*, commonly placed between the crown Mysticeti and the †Archaeoceti. Three recently named genera, in this fast-moving field of paleocetology, are not included in our analyses, but were placed phylogenetically in the original articles as more basal to the position of ^†^*Toipahautea*. The presumed baleen-bearing ^†^*Sitsqwayk cornishorum* is probably Chattian, and phylogenetically between †Eomysticetidae and †Aetiocetidae [25]. The toothed Rupelian ^†^*Coronodon havensteini* [3] is basal to †Aetiocetidae and one of the most basal Mysticeti, while the toothed Priabonian ^†^*Mystacodon selenensis* [4] is reportedly the most basal mysticete.

In both phylogenetic analyses of figure 11, ^†^*Toipahautea* is crown-ward of †Eomysticetidae, either in a polytomy with crown Mysticeti, or just below the crown. Hitherto, the oldest described fossil baleen whale close to or within crown Mysticeti is ^†^*Horopeta* (25–27 Ma), for which position varies depending on phylogenetic analyses (e.g. Tsai & Fordyce [7], fig. 14a: crown Mysticeti; 14b: stem mysticete). Consider also ^†^*Mauicetus parki* (approx. 24.7 Ma based on Sr/Sr isotopes), from the middle Otekaike Limestone at the ^†^*Toipahautea* and ^†^*Horopeta* type locality. ^†^*M. parki* has been proposed as an early balaenopteroid within the crown Mysticeti [16]. Our results in figure 11*a* also show ^†^*Mauicetus* as a crown Mysticeti, but ^†^*Mauicetus* is excluded from the crown Mysticeti in the alternative analysis of figure 11*b*. Inconsistent positions for taxa in a phylogeny reflect widely recognized factors including the number of individuals sampled, preservation, ontogenetic stage, character linkage and uncertain homology (discussed by Tsai & Fordyce [13]). It is also difficult to recognize true ancestry (see [10] on ancestry in a cladistic framework). Of note, because the holotype of ^†^*T. waitaki* is a physically immature individual, its (current) phylogenetic position would probably move more crown-ward if an adult individual were available to code. Elsewhere, we discussed the relationships between ontogeny and phylogeny in Mysticeti [26]. We suggest that, on balance, the relationships and ages of species of ^†^*Toipahautea*, ^†^*Horopeta, †Whakakai* and ^†^*Mauicetus* indicate that the MRCA of crown Mysticeti lived about the Early–Late Oligocene boundary or even in the Early Oligocene.

### 5.2. Early transition to fully baleen-bearing mysticetes

The presence of a sulcus for the superior alveolar artery on the ventral surface of the maxilla, and the thin laterally extensive maxilla, indicate the presence of baleen in ^†^*Toipahautea* (Ekdale *et al.* [22] discussed the osteological correlate for the presence of baleen). In addition, the edentulous mandible of ^†^*Toipahautea* is laterally and dorsoventrally bowed, and its coronoid process is well developed, and slightly laterally deflected, consistent with the condition in toothless Mysticeti. We interpret the 27.5 Ma ^†^*Toipahautea* as a baleen-bearing toothless mysticete, contrasting with the baleen-bearing †Eomysticetidae with remnant teeth.

Total loss of teeth in adults, and acquisition of baleen, are two independent major evolutionary events in mysticete evolution; they are regulated by different genes (e.g. [27–29]), probably allowing independent evolutionary results. Total tooth loss would involve multiple genes [30]. The fossil record is too patchy to say whether the evolutionary rate of tooth loss in Mysticeti was gradual or abrupt.

Two mainly Late Oligocene groups, †Aetiocetidae and †Eomysticetidae, may have had both teeth and baleen, but they represent quite different grades, or ecomorphs. †Aetiocetidae had well-developed teeth, but baleen is questionable [5,6,9]. Aetiocetids show evolutionary trends of tooth simplification from heterodonty to homodonty, and some increase in tooth number (polydonty in ^†^*Aetiocetus polydentatus*), but no indication of tooth loss. Conversely, †Eomysticetidae was nearly toothless with probably well-developed baleen racks in at least the posterior two-thirds of the rostrum [1,31].

The 27.5 Ma ^†^*Toipahautea*, and the slightly younger ^†^*Horopeta* and ^†^*Mauicetus*, are clearly distinct from the coeval †Aetiocetidae and †Eomysticetidae in terms of feeding apparatus, suggesting that the genetic sequence regulating tooth development had already actioned the total loss of dentition in adulthood. Another suite of genes independently led to the presence of fully functional baleen racks by the end of the Early Oligocene, concurrent with toothlessness.

^†^*Toipahautea*, †Aetiocetidae and †Eomysticetidae are coeval and morphologically disparate, without clear ancestor–descendant relationships. †Aetiocetids and †eomysticetids were speciose and short-lived clades—morpho-ecological experiments—of stem-Mysticeti that likely originated in the Early Oligocene and went extinct by the earliest Miocene [32]. †Aetiocetids and †eomysticetids are intriguing side-branches, and evolutionary dead ends, in mysticete evolution; they are distinct from the origin of crown Mysticeti, but show the morphological potential to bridge the transition between toothed and baleen-bearing forms. It largely remains uncertain where the true baleen-bearing mysticetes arose from toothed stem mysticetes such as †mystacodontids, †llanocetids, †mammalodontids, †aetiocetids, ^†^*Coronodon* and †eomysticetids. The latter groups show no compelling evidence of ancestor–descendant relationships leading to early toothless and baleen-bearing ^†^*Toipahautea*. For now, ^†^*Toipahautea* may serve as a best calibration point to consider the emergence of fully baleen-bearing and toothless mysticetes, or specifically the origin of crown Mysticeti.

### 5.3. Feeding mode in early Mysticeti

Coeval Oligocene mysticetes had diverse feeding behaviours, perhaps the greatest in mysticete history, in terms of: rostral form and kinesis; the presence (or loss), form and size of teeth; and development of baleen as indicated from rostral vascular sulci and possible jaw mechanics. The inferred feeding behaviours fall into these groups:

(1) ^†^*Toipahautea*, inferred to have proto-baleen and a long kinetic jaw and skull, shows a laterally curved coronoid process, suggesting that it may gulp-feed like ^†^*Horopeta* (discussed by Tsai & Fordyce [7]).
(2) Eomysticetids, with baleen and remnant teeth in long kinetic jaws, likely used another style of bulk-filtering—skim-feeding [33].
(3) Aetiocetids, with functional teeth in long akinetic jaws, were probably raptorial, with primitive bulk-filtering capability [9] and/or suction-feeding [5,6].
(4) The toothed mysticete ^†^*Janjucetus*, with functional teeth in short akinetic jaws, was likely raptorial [34].
(5) The toothed mysticete ^†^*Mammalodon*, with functional teeth in short kinetic jaws, was likely a bottom suction-feeder [35].
(6) The toothed mysticete ^†^*Coronodon*, with functional teeth in long akinetic jaws, was likely filter-feeding with help of teeth instead of baleen [3], and also see the interpretation from Hocking *et al.* [36].

Three scenarios have been proposed for the evolution of mysticete feeding from toothed to baleen-bearing:

(1) raptorial with proto-baleen to facilitate a primitive filtering-assisted style [9],
(2) raptorial with suction-assisted [5,6], and
(3) filter-feeding starting with teeth instead of baleen [3].

Highly disparate morphologies of coeval toothed mysticetes, such as ^†^*Coronodon, †Mammalodon, †Janjucetus* and aetiocetids from ^†^*Toipahautea*, the oldest known crown Mysticeti, suggest varying possible paths from teeth to baleen. The lack of ancestor–descendant relationships from toothed to baleen whales, such as ^†^*Toipahautea*, at present precludes determining which feeding mode was mainly employed while mysticetes had both teeth and baleen. However, consider that (i) ecomorphs of various feeding modes in the Oligocene mysticetes (possible gulp-feeding ^†^*Horopeta* and ^†^*Toipahautea*, skim-feeding eomysticetids, raptorial ^†^*Janjucetus*, suction-feeding ^†^*Mammalodon*, raptorial with possible primitive filter-feeding †aetiocetids and possible tooth-assisted filter-feeding ^†^*Coronodon*) and (ii) versatility of feeding strategy in extant mysticetes; for example, the gray whale *Eschrichtius robustus* can also perform gulp and skim-feeding as well as benthic suction-feeding [37] or ability to learn new feeding skills in balaenopteroids [38]. Perhaps, early mysticetes were like the gray whale or speciose balaenopterids and versatile in feeding strategies.

Given ^†^*Toipahautea* as the oldest known crown Mysticeti and a brief review summarized above regarding the feeding modes in Oligocene mysticetes, we, here, propose a scheme that some early mysticetes may have been generalists and opportunists instead of specializing in any particular forms of feeding strategies as they transitioned from toothed ancestry possibly specializing in raptorial to baleen-bearing, like ^†^*Toipahautea*, perhaps performing bulk-feeding, but not precluding the possibility of adopting new skills or approaches at feeding as seen in extant gray whales and balaenopterids. This scenario may help early mysticetes to avoid high ecological competition as they evolved from specialists (raptorial toothed forms) to generalists (transitional period), and then subsequently some lineages acquired their new niches to specialize in particular feeding strategies, such as skim-feeding in balaenids and gulp-feeding in balaenopteroids. This specialist–generalist–specialist hypothesis in mysticete evolution explains a possible awkward stage as mysticetes had both teeth and baleen during feeding.

## Supplementary Material

Tsai and Fordyce 2017 Toipahautea Dec31.nex

## Supplementary Material

Tsai and Fordyce 2017 Toipahautea Dec31.tnt

## Data accessibility

NEXUS and TNT files are available in the electronic supplementary material for the details of character/taxon dataset and re-run of phylogenetic analyses.
